# Fabrication of Surfactant-Dispersed HiPco Single-Walled Carbon Nanotube-Based Alginate Hydrogel Composites as Cellular Products

**DOI:** 10.3390/ijms20194802

**Published:** 2019-09-27

**Authors:** Fabian Alvarez-Primo, Shweta Anil Kumar, Felicia S. Manciu, Binata Joddar

**Affiliations:** 1 Inspired Materials & Stem-Cell Based Tissue Engineering Laboratory (IMSTEL), El Paso, TX 79902, USA; fpalvarez@miners.utep.edu (F.A.-P.); sanilkumar@miners.utep.edu (S.A.K.); 2 Department of Metallurgical, Materials and Biomedical Engineering, M201 Engineering, University of Texas at El Paso, 500 W University Avenue, El Paso, TX 79968, USA; 3 Department of Physics, University of Texas at El Paso, 500 W University Avenue, El Paso, TX 79968, USA; fsmanciu@utep.edu; 4 Border Biomedical Research Center, University of Texas at El Paso, 500 W University Avenue, El Paso, TX 79968, USA

**Keywords:** biocompatible, composites, surfactants, dispersion, anionic, cationic, steric

## Abstract

In this study, we designed, synthesized, and characterized ultrahigh purity single-walled carbon nanotube (SWCNT)-alginate hydrogel composites. Among the parameters of importance in the formation of an alginate-based hydrogel composite with single-walled carbon nanotubes, are their varying degrees of purity, their particulate agglomeration and their dose-dependent correlation to cell viability, all of which have an impact on the resultant composite’s efficiency and effectiveness towards cell-therapy. To promote their homogenous dispersion by preventing agglomeration of the SWCNT, three different surfactants-sodium dodecyl sulfate (SDS-anionic), cetyltrimethylammonium bromide (CTAB-cationic), and Pluronic F108 (nonionic)-were utilized. After mixing of the SWCNT-surfactant with alginate, the mixtures were cross-linked using divalent calcium ions and characterized using Raman spectroscopy. Rheometric analysis showed an increase in complex viscosity, loss, and storage moduli of the SWCNT composite gels in comparison with pure alginate gels. Scanning electron microscopy revealed the presence of a well-distributed porous structure, and all SWCNT-gel composites depicted enhanced electrical conductivity with respect to alginate gels. To characterize their biocompatibility, cardiomyocytes were cultured atop these SWCNT-gels. Results comprehensively implied that Pluronic F108 was most efficient in preventing agglomeration of the SWCNTs in the alginate matrix, leading to a stable scaffold formation without posing any toxicity to the cells.

## 1. Introduction

Alginate, as a naturally occurring polymeric material, has exhibited excellent compatibility for biomedical purposes. Alginate is commonly used in the form of a hydrogel in biomedical applications, comprising wound healing, tissue engineering, and drug delivery [1]. Hydrogels are three-dimensionally cross-linked networks composed of hydrophilic polymers with high water content [1].

The use of alginate hydrogels in tissue engineering and regenerative medicine is not uncommon, placing it among the preferred biomaterials for the culturing of cells, as well as their delivery, due to its similarity to the extracellular matrix environment (ECM) [2]. As hydrogels require cross-linking [3,4] to improve their physicochemical properties and retain structural fidelity, the ionic gelation process can be difficult to control due to the concentration of calcium ions present at the time of reaction. This may result in nonuniform and inferior mechanical properties in the resultant alginate hydrogels, which cannot be used as tissue scaffolds due to their dissimilarities with mechanical characteristics of physiological tissues [4]. In fact, a recent study has shown that the physical properties of alginate hydrogels produced in combination with carbon nanomaterials depend on the chemical route followed during the gelation process [5,6,7].

In previous research, Joddar et al. developed and characterized multiwall CNT embedded alginate hydrogels and studied the effects of increasing concentration of CNT on resultant composite’s characteristics and their cell compatibility [8]. The results showed that 1 mg/mL of multiwall CNT lead to the formation of most stable composite hydrogels in comparison with gels made with higher doses of CNTs. All the CNT-embedded alginate gel composites depicted enhanced mechanical characteristics in comparison to the pristine alginate hydrogels. Moreover, the 1 mg/mL multiwall CNT-alginate gels allowed enhanced cell proliferation and migration accompanied by ECM secretion in comparison with gels made with higher doses of CNTs. As it is widely known and accepted that CNTs can be cytotoxic to physiological tissues, our goal was to pursue a line of research seeking to lower the dose of CNTs; furthermore, by promoting their enhanced dispersion, keeping the mechanical and physical characteristics of these newly developed CNT-gel composites intact. In this study, we used high-pressure carbon monoxide (HiPco) process created single-walled carbon nanotubes (SWCNT) processed from the gas-phase reaction of iron carbonyl with high-pressure carbon monoxide gas [9]. These SWCNT produced by the HiPco process at RICE University [9] are of high purity (≥ 90%) and fibrous in nature, which enables easy dispersions in organic solvents thereby reducing the overall dose of CNT used for making composite products. SWCNTs were mixed into various surfactants, namely, sodium dodecyl sulfate (SDS-anionic), cetyltrimethylammonium bromide (CTAB-cationic), and Pluronic F108 (PF108-nonionic) to promote their homogenous dispersion with extremely low doses of CNTs, in comparison with our previous study [8]. As CNTs are known to form clusters readily due to Van der Waals interactions, it difficult to keep them homogenously dispersed through the matrix. Therefore, our objective was to prevent such natural interactions by forcing them to react with the various surfactants, namely with SDS in an anionic interaction, with CTAB in a cationic manner and with PF108 by way of steric hindrance [10]. These SWCNT-surfactant solutions were then mixed with the alginate solution, and the mixture cross-linked with divalent calcium ions to promote the formation of SWCNT-embedded alginate gel composites. We hypothesized that the use of various surfactants to promote uniform SWCNT particle dispersion by different interaction mechanisms would enable comparison of the resultant SWCNT-alginate composites based on their physical and rheological characteristics along with cell compatibility. The results will contribute to establishing a novel method of making SWCNT-alginate composites and help optimize the use of a dispersion agent for making electrically conductive substrates for cell therapy and other applications.

## 2. Results

### 2.1. Fabrication of SWCNT-Alginate Composites

The resulting SWCNT-surfactant solutions were prepared as described in Section 4.2. The proper concentration of SWCNT for incorporation into the alginate hydrogels was determined in a previous study, demonstrating an upper range in which gelation was achieved [10]. Briefly, this upper limit was shown to be at a concentration of 30 parts per million (ppm) [11,12] with any further increases resulting in the enhancement of cytotoxicity of resultant composites. For the purpose of this study, we chose a lower limit of 25 ppm, to maintain a much higher stability of the SWCNT-embedded hydrogels. Furthermore, additional calculations were done to accurately obtain the 25 ppm desired concentration, due to a property known as mass percent conversion (MPC), which has been previously reported by Moore at al. in the suspensions analyzed in this study [10]. From this study by Moore et al., guidance was obtained on utilizing a parameter called mass percent conversion or MPC, which refers to the measure of the surfactant or polymer’s ability to suspend the SWCNT, left to decant in comparison to the original concentration. The reported values of MPC for SWCNT-surfactant are 3.3%, 5.1%, and 8.7% for SDS, CTAB, and PF108, respectively [10]. Because of the differing solubility of the SWCNTs in the various surfactants resulting from their unique interactions, the resultant solubilized concentration of these SWCNT in these solutions was significantly lower than 2 mg/mL.

These surfactant dispersed SWCNT solutions with varying MPC values were then added to a 4% (*w*/*v*) sodium alginate in deionized (DI) water and cross-linked with a 0.25 M calcium chloride (CaCl_2_) solution [8,13]. Based on the previously reported MPC values, to achieve a 25 ppm (0.025 mg/mL) concentration for a gel sample containing SWCNT in SDS accurately, 1.136 mL was added to the alginate solution and cross-linked with CaCl_2_ to obtain a composite containing a 25 ppm concentration of SWCNT. For CTAB, exactly 0.735 mL was added, and for PF108, 0.431 mL was added to alginate solution and cross-linked with CaCl_2_ to obtain a composite containing 25 ppm or 0.025 mg/mL concentration of SWCNT, respectively. Once casted, these SWCNT-gel composites are stable for one month when stored at 4 °C.

As shown in Supplementary Figure S1, images of the four samples representing control alginate gels (Control) and gels with SWCNT-surfactants depicted as (SDS) for SDS; (PF108) for PF108 and (CTAB) for CTAB were taken to visually compare among each other. Our controls showed a transparent appearance, in contrast to SWCNT-surfactant loaded samples. As SDS had the lowest MPC among the three surfactants used, the greatest volume of SWCNT-SDS was required to achieve the 25 ppm, making it the least light permissive among all samples shown. CTAB had a relatively higher MPC than SDS, which required a smaller volume of SWCNT-surfactant to achieve the desired concentration of 25 ppm. However, particulate clustering was more noticeable in this set of samples. Finally, PF108 demonstrated the higher MPC, which required a minimum volume to achieve the desired concentration of 25 ppm, resulting in a composite gel that resembled controls.

In this study, a series of surfactants, namely, SDS (anionic), CTAB (cationic), and PF108 (nonionic/steric), were tested for their ability to suspend SWCNT. Among the three, the nonionic surfactant or PF108′s ability to suspend SWCNT appeared to be most effective due mostly to the size of the hydrophilic group, with higher molecular weights suspending more nanotube material because of enhanced steric stabilization with longer polymeric groups [10]. In contrast, the anionic or cationic surfactants were not as efficient in dispersing the SWCNT and exhibited clustering in the composite gels, as seen in Supplementary Figure S1. PF108 was used at the lowest volume of the three surfactants owing to its high MPC and demonstrated no visible signs of clustering of the SWCNT in the composite gels.

### 2.2. Raman Spectroscopic Characterization of SWCNT-Alginate Composites

**Figure 1:**
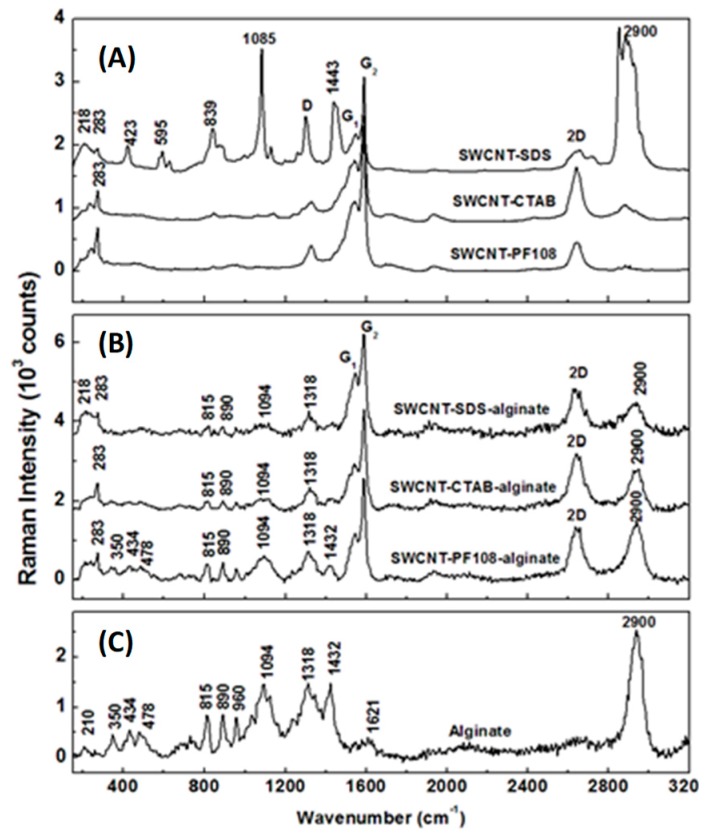
Raman spectra of (**A**) single-walled carbon nanotube (SWCNT) functionalized with different surfactants, (**B**) SWCNT-surfactant gel composites, and (**C**) alginate hydrogel, as labeled. For easier visualization, the Raman spectra in (**A**) and (**B**) are vertically translated.

To ensure the incorporation of SWCNT within gels and that their retention in a dispersed form by the addition of surfactants, Raman spectroscopy was performed, and the results are presented in Figure 1A–C. For cross-checking and comparison purposes, as well as for an accurate assignment of the observed Raman vibrations, only the Raman spectra of SWCNT-surfactant mixtures are shown in Figure 1B and only that of alginate hydrogel in Figure 1C. All the Raman spectra were appropriately labeled, and those in Figure 1A,B are vertically translated for easier visualization.

It is well established that the intensity and broadness of the characteristic Raman vibrational lines at 1318 cm^−1^ (D band) and around 1580 cm^−1^ (G band) are a direct estimation of carbon-based sample quality [14,15,16,17,18]. Although the D mode, also known as the disordered carbon line, is an inherent Raman feature of SWCNT and related to symmetry lowering or finite-size effects, the G line of graphite commonly splits into a broad peak G_1_ at 1550 cm^−1^ and a narrow feature G_2_ at 1580 cm^−1^. The sharpness of the G_2_ peak is also a direct indication of potentially unwanted SWCNT bundling [17]. Furthermore, through analysis of Raman vibrational lines associated with the radial breathing modes (RBMs), validation of a metallic or a semiconducting content of SWCNT can be achieved [17,18]. The presence of all these Raman features in the current spectra allows us to perform an inclusive analysis, as well as confirming the employed SWCNT is of high quality.

A strong presence of the SDS surfactant is observed in Figure 1A, and the corresponding SWCNT-SDS Raman spectrum, with vibrational lines around 423, 595, 839, 1085, 1443, and 2900 cm^−1^. More importantly, the lower intensity of the G_2_ peak seen in this spectrum, in comparison with the intensities of this line in the Raman spectra of SWCNT-CTAB and SWCNT-PF108, demonstrates a potential clustering of SWCNT in SDS. This bundling is also implied by the weak Raman feature around 218 cm^−1^, which is absent in the other two spectra. These remarks confirm a lower solubility of SWCNT in SDS than that in CTAB or PF108, corroborating with the visual appearances of the samples in Supplementary Figure S1. The RMB Raman peak at 283 cm^−1^ validates the semiconducting characteristic of SWNTs used in this study [17,18]. The higher intensity of the D band observed in the SWCNT-SDS Raman spectrum, compared with the Raman spectra of SWCNT-CTAB and SWCNT-PF108, also indicates a higher amount of defects and bending of the CNT in the former case.

Investigations of SWCNT-surfactant-gel composites shown in Figure 1B reveal a more effective dispersion of SWCNT in alginate hydrogel when CTAB and PF108 are used than in the case of SDS employment. Besides being expected from our analysis of Raman spectra presented in Figure 1A, this affirmation is also supported by more noticeable Raman vibrations attributed to alginate in the SWCNT-CTAB-alginate and SWCNT-PF108-alginate spectra, such as the peaks at 210, 350, 434, 478, 815, 890, 960, 1094, 1318, 1432, 1621, and 2900 cm^−1^ (see Figure 1C). The overlapping of alginate vibration at 1318 cm^−1^ with the D band prevents further examination of other induced defects to SWCNT that might occur during the SWCNT-surfactant embedment within the gel. Thus, among the three surfactants, the PF108 (steric), followed closely by the CTAB (cationic), reveal higher ionic stability than SDS (anionic) in the cross-linking process of forming the SWCNT-surfactant gel composites.

### 2.3. Near-Infrared (NIR) Fluorescence Microscopy of SWCNT-Alginate Composites

**Figure 2:**
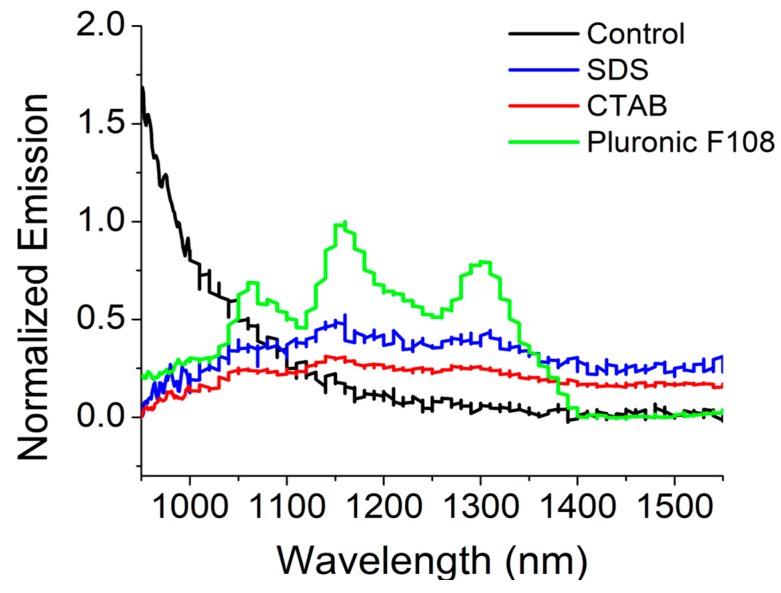
Near-infrared (NIR) photoluminescence spectra of different surfactant/SWCNT-based hydrogels.

HiPCo SWCNT are known to emit near-infrared (NIR) photoluminescence (PL) in the long wavelength region [19]. The retention of pristine SWCNT single-walled carbon nanotubes dispersed via surfactants and encapsulated within alginate gel composites was confirmed using NIR PL intrinsically emitted by the nanotubes. When exposed to 658 nm for excitation, the SWCNT-CTAB and PF108 composite gels exhibited some characteristic nanotube PL spectra indicating localization of nanotubes in these samples, compared with the other gel samples (Figure 2). These results imply that the SWCNTs were effectively dispersed and prevented from agglomeration by PF108 and CTAB, but not by SDS. NIR PL measurements of the other gels appeared to display weak emission from some individualized CNT within the gels. The sample prepared from PF108, in particular, showed individual NIR emission peaks similar to those seen in CNT surfactant dispersions of semiconducting nanotubes [19]. These observations may be attributed to the fundamental interactions of the SWCNT-surfactant systems with sodium alginate and cross-linking mechanism via calcium chloride [8], as discussed later in Section 3.

### 2.4. Rheological Characterization of SWCNT-Alginate Composites

**Figure 3:**
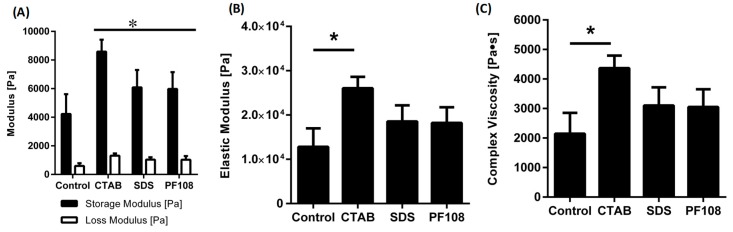
Rheological properties of different surfactant/SWCNT-based hydrogels. * indicates statistically significant difference (*p* < 0.05) in comparison with controls. Only the CTAB samples appeared to have significantly increased values for elastic modulus and complex viscosity in comparison with controls, as shown in panels (**B**) and (**C**). All SWCNT-el samples depicted a significant increase in Storage and Loss Moduli, in comparison with controls (**A**).

The rheological analysis was performed with the objective of studying the viscoelastic properties of different hydrogel compositions (Figure 3) [8]. All the samples were subjected to strain sweep tests to establish the linear viscoelastic region where both the storage and loss moduli were independent of the applied strain, as done previously [20,21]. In all the cases, the storage moduli were significantly higher than the loss moduli indicating that the synthesized gels were all chemically cross-linked (Figure 3A). It was also observed that the incorporation of SWCNT into the hydrogels with any of the three surfactants increased the elastic moduli (Figure 3B) and complex viscosity (Figure 3C), as opposed to the control sample. Further, the mechanical properties of the CTAB infused hydrogels were found to be superior to those of the hydrogels with SDS and PF108 (Figure 3A,B). The electrostatic interaction between positively charged surfactant, CTAB, and anionic groups present in the sodium alginate caused an overall stiffening of the matrix and enhancement of all rheological properties [22].

### 2.5. Electrical Characterization and Conductivity of SWCNT-Alginate Composites

**Figure 4:**
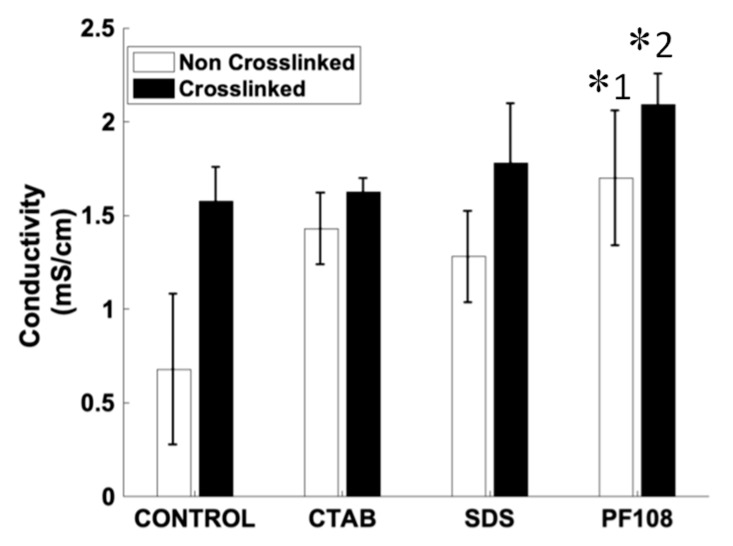
Conductivity values of different surfactant/SWCNT-based hydrogels. * indicates statistically significant difference (*p* < 0.05) for non-cross-linked (*1) and cross-linked samples (*2) of PF108, in comparison with controls.

It is known that Ca^2+^ ions used in the cross-linking of alginate gel systems can influence the magnitude of the observed conductivity [13]. Therefore, a comparison was made between pre- and post-cross-linked SWCNT-surfactant dispersions in alginate gels. Electrical conductivity (Figure 4) was studied pre- and post-cross-linking of the SWCNT-surfactant dispersions in alginate gels, to understand how the mode of dispersion (SDS-anionic, CTAB-cationic, and PF108-steric) of the SWCNT influenced the increase in electrical properties in comparison to control alginate gels. Among the non-cross-linked samples, the SWCNT-PF108 showed the maximum values compared to other SWCNT-surfactant systems (as indicated by *1).

As PF108 was used at the lowest volume of the three surfactants, owing to its high MPC and steric nature, it allowed for a better dispersion of the SWCNT compared to the other surfactant systems used. Proportionately, the conductivity values for the SWCNT-PF108 was highest among all cross-linked samples as well (as indicated by *2). Among the cross-linked systems, a consistent increase in conductivity occurred across all samples, including the pristine alginate gels, due to the presence of Ca^2+^ ions. It is interesting to note that these results are in range with conductivity values reported for actual physiological tissues in other published works [23,24]. In fact, the SWCNT-PF108 gel composite yielded a value, which was almost double the electrical conductivity reported from actual physiological tissues [23,24].

### 2.6. Scanning Electron Microscopy (SEM) of SWCNT-Alginate Composites

**Figure 5:**
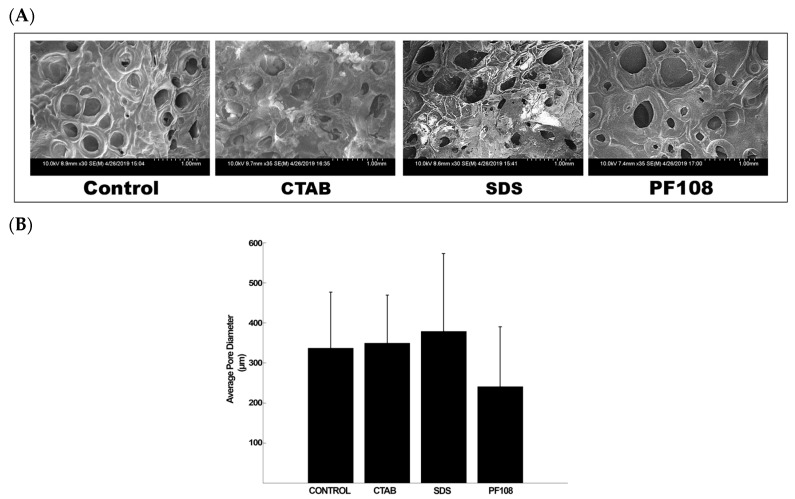
Characteristic SEM images (**A**) and average pore size distribution (**B**) of different surfactant/SWCNT-based hydrogels.

In this study, composite scaffolds of alginate and SWCNT were fabricated by cross-linking sodium alginate with calcium chloride, which produces micropores during the gelation process [18]. To confirm that the introduction of SWCNT-surfactant into this alginate gel system did not disorder this natural pore formation process, SEM imaging was conducted. In general, all SWCNT-surfactant gel composites demonstrated a stable internal structure with homogenously distributed pores and no evidence of stress cracking in any of the samples imaged (Figure 5A) when compared with alginate gel controls (Control). However, the SWCNT-PF108 composite gels exhibited the smallest average pore diameter of 241.36 µm, among all samples (Figure 5B). More characteristic SEM images for all samples and controls are included in Supplementary Figure S2. These additional images demonstrated the rough surface of the composites that maintained “crater-like” surfaces. However, these surfaces became shallower for the SWCNT-CTAB and -SDS-based composite gels in comparison with the other cases.

### 2.7. Swelling and Degradation

**Figure 6:**
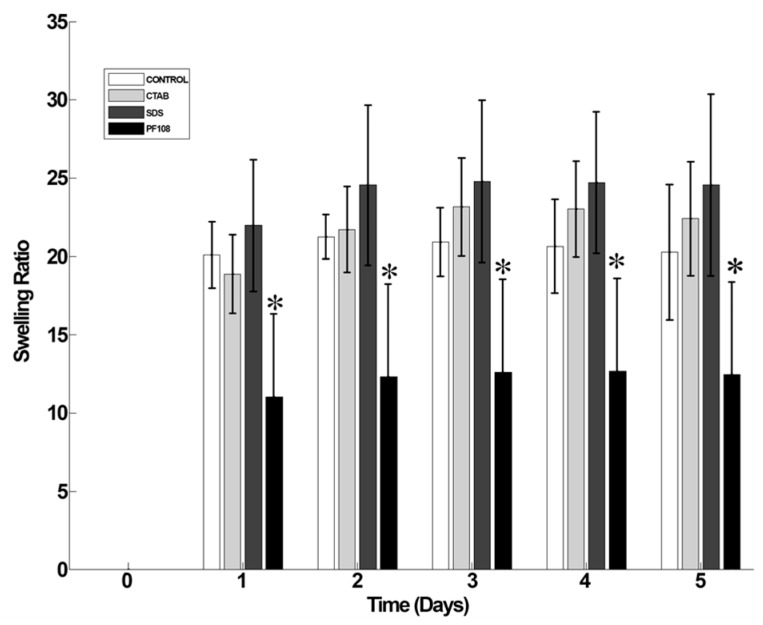
Swelling analysis of different surfactant/SWCNT-based hydrogels. * indicates statistically significant difference (*p* < 0.05) in comparison with controls.

The swelling kinetics and behavior of all SWCNT-surfactant gel composites were analyzed and depicted in Figure 6. From this data, we conclude that the addition of SWCNT-surfactants within alginate did not adversely affect the stability of the cross-linked alginate gels [8]. Among the SWCNT-surfactant gel composites, the PF108 dispersed gel composites showed the least amount of swelling compared to other systems making it the most stable system compared to all other cases (as indicated by *). However, the SWCNT-SDS gels and the SWCNT-CTAB gels showed a higher degree of swelling when compared to SWCNT-PF108 set of gel composites. This could be due to SWCNT-PF108 gels having the smallest average pore diameter of all systems analyzed and this case being the most stable in terms of SWCNT homogenous dispersion by the PF108. All SWCNT-surfactant gel composite systems reached an equilibrium swelling degree at two days’ time, implying stability of the SWCNT-surfactant embedded gel systems similar to the alginate control gels.

### 2.8. Cell Culture and Live/Dead Assay

**Figure 7:**
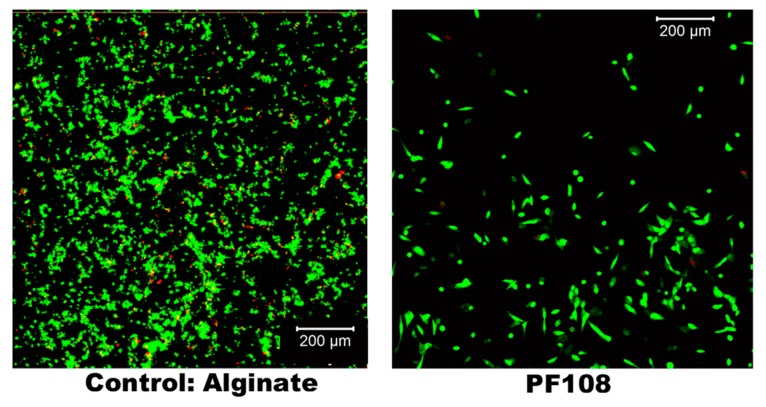
Representative live-dead assay stained images of cells cultured in SWCNT-PF108 composites (right) and pristine alginate gels (left).

After 72 h, the cultures were analyzed using a Live/Dead assay with Calcein AM (green) that stained the live cells and Ethidium homodimer (red) that stained the dead cells, respectively [20]. Results depicting the cytotoxicity of these SWCNT-surfactant gel composites are shown in Figure 7. Among the SWCNT-surfactant gel composites, viable cells were detected only in the SWCNT-PF108 gels. These results are in agreement with other’s published study that Pluronic appears to be highly biocompatible if used at low concentration [25]. The number of live cells in the SWCNT-PF108 gels was 80% ± 5% in comparison with alginate gels, which showed about 92% ± 8% live cells. These sets of values were not statistically different. Neither live nor dead cells were detected in the other SWCNT-surfactant gels. Although we did not perform any follow-up experiments to determine the reason for this observation, it could be attributed to the enhanced cytotoxicity associated with the surfactants, SDS and CTAB [10].

Table 1 depicts a comprehensive summary of all results obtained in this study wherein the behavior of SWCNT-surfactants systems in alginate was compared to alginate only.

## 3. Discussion

The different methods developed to fabricate scaffolds with pore architectures developed designed in the last decade include gas foaming [26], sintering fiber meshes [27], solvent casting [28], polymerization in solution [29], electrospinning [30], 3D printing [31], and 3D bioprinting of scaffold with cells [32]. One of the popular strategies adopted for the creation of engineered tissues is the culture of isolated cells on three-dimensional scaffolds along with permitting conditions that lead to the development into a functional tissue. The scaffolds can be fabricated from synthetic polymers or from natural materials, such as alginate, collagen, or gelatin, to provide the biomechanical support needed by the growing cells. As the cells grow and differentiate on the scaffold, they secrete their own ECM, which supports the growth and maturation of a functional tissue, in vitro.

The feasibility of engineered functional cardiac muscle has been demonstrated by many research groups, including us [20,33]. However, most study outcomes have led to cardiac muscle constructs with a number of inadequacies that limit their effectiveness in vitro and in vivo applications. Unlike native cardiac muscle that consists of fibers with a defined alignment, the cells in engineered constructs exhibit random alignment and poor degree of functional maturation [34]. Thus, its usefulness as a cellular implant for replacement therapy is limited. Therefore, a need exists for techniques allowing the creation of robust physiological tissues in vitro, leading to enhanced integration and functionality of transplanted tissues in vivo [11]. To engineer functionally mature cardiac tissue constructs, it is essential to have them in scaffolds such as hydrogels, which supports cell retention, survival, and integration of host cells. However, hydrogels casted from commonly used biopolymers such as gelatin, alginate, or collagen are electrically nonconductive, and they must be infused with another secondary conductive material to make them serve the dual functions of being biocompatible and electrically conductive. The intimate contact between the cultured cells and the electrically conducting component in the tissue scaffold then acts like a biological electrode and dramatically lowers the energy threshold required to induce action potentials or for electrically pacing the cells, in the case of a cardiac construct. Electrically stimulated pacing of cultured cardiomyocytes on such biocompatible and electrically conductive scaffolds serves as an experimentally convenient and physiologically relevant model of cardiac tissue in vitro [34]. Electrical stimulation of cardiac cells leads to enhancement in the expression of connexins in myocardial cells, which in turn, form gap junctions [34]. In our previously published research study [8], we synthesized functionalized multiwall CNT-alginate composite gels with distinctively different mechanical, physical, and biological characteristics in comparison to alginate alone. The resultant MWCNT-alginate gels were porous and showed significantly less degradation in vitro, compared to alginate alone. In vitro cell studies showed enhanced HeLa cell adhesion and proliferation on the MWCNT-alginate compared to alginate. Among all the MWCNT-alginates, the 1 mg/mL gels showed significantly higher stiffness compared to all other cases. These results provided an important basis for the development of the MWCNT-alginates as novel substrates for cell culture applications, cell therapy, and tissue engineering. However, 1 mg/mL of CNTs in the alginate may still be cytotoxic to cardiac cells [35]. Therefore, our goal was to seek approaches to lower the overall concentration of CNTs encapsulated within the alginate gels.

A large number of surfactants have been studied for dispersion of SWCNTs. The surfactant molecules are mostly amphiphilic in nature, and during the adsorption process, the hydrophilic ends of the surfactant interact with water molecules while the hydrophobic ends interact with the hydrophobic surface of the SWCNTs, thus stabilizing them in aqueous medium [36]. In this study, this was achieved by the use of dispersion agents such as CTAB, SDS, and PF108, which interacted with the SWCNTs by various mechanisms. At high surfactant-to-CNT ratios, the surfactant can stabilize the SWNTs electrostatically or sterically, to overcome the attractive van der Waals forces between them. However, at low surfactant concentration, there is insufficient coverage on the surface by surfactant molecules and hydrophobic attraction between the SWCNTs themselves, which leads to the agglomeration or flocculation of the SWCNT bundles [36]. In this study, all surfactants were used in the range depicting a high surfactant-to-CNT ratio, as guided by published literature [36]. Among the surfactants used, CTAB is cationic, which causes the CNTs to acquire a positive surface charge with a surface potential that depends on the surfactant-to-CNT ratio. For SDS, which, although is anionic in nature and provides a high zeta-potential to the SWCNTs particles affording effective dispersion, they failed to sufficiently surface adsorb and stabilize these CNTs in water [36]. However, PF108 was estimated to be the most effective system for promoting SWCNT dispersion based on results obtained in this study. PF108 is amphiphilic and does not confer any specific charge to these SWCNTs. Our results are in agreement with previously published reports, where it was clarified that when CNTs are added to Pluronic, first, the bundles disaggregate, kinetically driven by the energy supplied to the system; second, they disperse via surfactant adsorption, thermodynamically driven by the surfactant concentration [25]. As the CNTs are simply surface adsorbed without any chemical interaction, they do not oppose the strong ionic bonding of the alginate via a calcium-based cross-linking mechanism, as shown by published works [1,4,5,7,8,13,37,38,39,40,41]. Furthermore, PF108, being a polymer, exhibits a long flexible chain-like organization in water that allows physical entanglement and entrapment of the surface adsorbed SWCNTs within the alginate gel due to mechanical mixing [42]. Therefore, the SWCNT-PF108 samples were the most stable composites in comparison to the other systems. In comparison, for the CTAB-based system, the SWCNTs become positively charged, affording a primary covalent bonding of the CNTs with the anionic moieties in alginate, leading to enhancement of rheological properties. However, this interaction reduces the overall amount of free anionic moieties in alginate available for ionic bonding via the calcium-based cross-linking mechanism leading to the deterioration of electrical conductivity. In contrast, for the SDS based system, the SWCNTs are negatively charged that sterically hinders chemical cross-linking of the CNTs with the anionic moieties in alginate leading to the deterioration of rheological properties due to flocculation of SWCNTs with time, although this did not hinder the ionic bonding of the alginate via a calcium-based cross-linking mechanism. However, this interaction did promote the dispersed SWCNTs to be freely available as isolated structures for the exhibition of other characteristic properties, such as electrical conductivity. Based on all of our experimental results, the stability of the SWCNT-gel composites prepared follows the trend:

$$\frac{\mathrm{SDS}<\mathrm{CTAB}<\mathrm{PF}108}{Stability\to }$$

Adoption of these dispersants allowed the overall dose of CNTs to be significantly lowered in comparison to our previously published works [8]. However, this reduction in the dose of CNT did not lead to a compromise in the resultant composite gels’ mechanical properties in comparison to our previous study [8]. This was because the dose of SWCNTs was lowered in accordance to published study guidelines, to achieve a high surfactant-to-CNT ratio necessary for making stable homogenous dispersions of SWCNTs in dispersants used in this study [36]. Furthermore, the newly synthesized set of SWCNT-surfactant dispersed gels also retained structural integrity as shown by the swelling analysis. Interestingly, in this study, we found that the surfactant dispersed SWCNTs exhibited NIR photoluminescence spectra, which are characteristic of these CNT materials. In addition, all cross-linked gels possessed electrical conductivity and showed an overall stable scaffold structure devoid of stress cracking when observed under SEM, in comparison to our previous study. Biocompatibility assessment proved that SWCNT-PF108 served as the most cytocompatible scaffold in comparison with other SWCNT-alginate samples. This implies that PF108 was the only cytocompatible dispersion agent among the three types of solvents used for dispersing CNTs, as it was introduced at the least amount (volume) in comparison to the other systems. To continue using the CTAB and SDS based systems for cell therapy, future studies must adopt lower quantities of these surfactants to be incorporated and introduce sonication or other means of effective mixing of the SWCNT-surfactant mixture within the alginate, prior to calcium cross-linking.

In a previously published study by Moore et al., several anionic, nonionic, and cationic surfactants were adopted to suspend and disperse SWCNTs [10]. Based on their published outline, we adopted three different surfactants, namely SDS, CTAB, and PF108, to disperse the SWCNTs in this study. Of these, PF108 showed maximum effectiveness as a dispersing agent when characterizing the properties of the resultant composite gels. Although PF108-suspended nanotubes have many applications, the use of other surfactant and polymer systems can significantly increase the applications of suspended nanotubes. For example, in the biomedical field, poly(ethylene oxide) is the preferred solubilizing polymer, and future studies will address the utility of this and other biocompatible electrically conductive polymers, such as poly(pyrrole) and poly(thiophene), or establish if poly(aniline) can be used as a dispersion agents for SWCNTs for cell and tissue culture applications [10,43]. Other studies have shown the surfactant, sodium dodecylbenzene sulfonate (SDBS) to demonstrate superior dispersion efficiency and exhibit all indicators of isolated, individual SWCNTs [36]. This research will lead to a fabrication of a new generation of electrically conductive hydrogels that can be 3D printed to promote either homogenous dispersion of SWCNT or their intended random dispersion to mimic anisotropic properties of biological tissues. On the other hand, SWCNT-surfactant alginate gels can be used as bioimaging probes based on the properties of SWCNT, which leads to the generation of longer wavelength photoluminescence (> 1000 nm) [44]. As CNTs do not contain heavy metals, they provide a safety advantage over the quantum dots used as imaging probes [45]. Future technological advances can be targeted to make biodegradable SWCNT-alginate gels that are required to endow CNTs with the specific tissue targetability required for use in a broader spread of biomedical applications.

## 4. Materials and Methods 

### 4.1. Experimental

HiPCO SWCNT (HR32-009) were obtained from NanoIntegris (Boisbriand, QC, Canada) and purified by a previously reported method [46]. Anhydrous calcium chloride (CaCl_2_) was obtained from Sigma-Aldrich (St. Louis, MO, USA). Phosphate buffered saline (PBS) buffer solution (1×) and Dulbecco’s Modified Eagle’s Medium (DMEM) were obtained from Gibco (Invitrogen, Carlsbad, CA, USA). Medium Viscosity sodium alginate was acquired from MP Biomedicals, LLC (Solon, OH, USA). The three surfactants (SDS, CTAB, and PF108) used to suspend the HiPco SWCNT were acquired from Sigma-Aldrich (St. Louis, MO, USA), Acros Organics (Morris, NJ, USA), and BASF (Florham Park, NJ, USA), respectively.

### 4.2. Fabrication of SWCNT-Alginate Composites

Three different surfactant solutions were utilized to determine which was most efficient for diminishing the van der Waals interaction between the CNTs. To achieve this, ~20 mg of HiPco SWCNT was added to a 1% wt. of surfactant solutions (CTAB, SDS, or Pluronic F108) for a final concentration of 2 mg/mL for all surfactants used as a means to disperse the SWCNT. These surfactant-dispersed SWCNTs were then subjected to ultrasonication (Cole-Parmer 8891, 42 kHz, 20 min, 25 °C) followed by centrifugation (Centrisart A-14, Sartorius) at 16,000 g for 30 min, and the clear supernatant collected for the study. These surfactant dispersed SWCNT solutions were then added to a premade aqueous solution of 4% (*w*/*v*) sodium alginate in DI water, and cross-linked with a 0.25 M CaCl_2_ [8]. Once cross-linked, these SWCNT-gel composites were considered stable for one month when stored at 4 °C. An 8 mm biopsy punch (Acuderm Inc., ThermoFisher Sci., Waltham, MA, USA) was used to obtain disc-shaped samples for all gels to enable comparison between samples of equal shapes and sizes. Digital images of these samples were acquired using an EVOS XL Core microscope (ThermoFisher Sci., Waltham, MA, USA)

### 4.3. Raman Spectroscopic Characterization of SWCNT-Alginate Composites

Raman measurements were acquired at ambient conditions in a backscattering geometry with an alpha 300R WITec system (WITec GmbH, Ulm, Germany). A 532 nm excitation of a frequency-doubled neodymium-doped yttrium-aluminum-garnet (Nd: YAG) laser that was kept at a low power output of 5 mW, and a 20 × objective lens with a numerical aperture of 0.4, were used for data acquisition. The Raman signal was detected by a 1024 × 127 pixel Peltier-cooled CCD camera with a spectral resolution of four wavenumbers. Accumulation of 20 Raman spectra, each spectrum recorded for 500 ms, with an overall Raman acquisition time of 10 s per sample was employed. Appropriate background subtractions were performed for all Raman spectra [47].

### 4.4. Near-Infrared (NIR) fluorescence Microscopy of SWCNT-Alginate Composites

The near IR (NIR) photoluminescence of each gel was measured using an Applied NanoFluorescence NanoSpectralyzer with a 658 nm laser excitation and 4000 ms integration time. Three scans were averaged together for each sample. Samples were prepared by adding a small piece of each gel to a 1 cm quartz cuvette and surrounding it with water.

### 4.5. Rheological Characterization of SWCNT-Alginate Composites

Gels for rheometry were formulated as described earlier and were cut using a biopsy punch (~1 mm deep, 8 mm diameter). The gels were pre-swollen in 1 × PBS prior to testing. Oscillatory shear stress rheometry was performed (1% strain, 0.5–50 Hz) using an Anton-Paar MCR101 rheometer (Anton-Paar, Graz, Austria) with an 8 mm parallel plate geometry. The strain and frequency range were estimated within the linear viscoelastic range of the gels through frequency sweeps. Elastic modulus was calculated using complex shear modulus with storage and loss moduli, and the complex viscosity was measured at 1.99 Hz for all samples, as done earlier [48].

### 4.6. Electrical Characterization and Conductivity of SWCNT-Alginate Composites

Gels for electrical conductivity measurement were cut using a biopsy punch (~1 mm deep, 8 mm diameter) and were pre-swollen in 1 × PBS for 12 h before testing. These swollen gels were then transferred to 50 mL centrifuge tubes for the testing. Measurements were done using the Mettler Toledo Seven Compact Duo S213 Benchtop pH/Conductivity Meter (Cole-Parmer, Vernon Hills, IL, USA). For each sample, the conductivity probe was immersed within the sample at the bottom of the tube, such that the tip was completely covered by the gel. In this position, the probe was held for at least 10 s until the reading was stabilized. Three repeats were conducted for each sample present.

### 4.7. Scanning Electron Microscopy of SWCNT-Alginate Composites

Cross-sectional images of the dried-gels were acquired using SEM, following published procedures [8]. For sample preparation, for imaging of the cross-sections, uniform-sized gels were made and freeze-dried and sputter-coated with gold/palladium (2–3 min) in a sputter coater (Gatan Model 682 Precision etching coating system, Pleasantown, CA, USA) and visualized using SEM (S-4800, Hitachi, Japan) at voltages of 12 kV at varying magnifications. Resultant images obtained were analyzed using Image J to determine their average pore diameter (µm) and its variation across samples.

### 4.8. Swelling and Degradation

To account for the hydration parameters of the CNT-alginate composite hydrogel structure leading to swelling, gels were allowed to swell to equilibrium for five days in Dulbecco’s Modified Eagle’s Medium (DMEM, pH = 7, 25 °C) following published protocols [8]. This process was conducted to identify the point in time at which swelling ratio and weight were found to be constant.

Samples were air-dried, weighed (W0), and then immersed in DMEM, and the swelled weight was recorded periodically (Wt) after every 24 h for five days. The swelling ratio was calculated using the following equation (1),

(1) Ds=(Wt−Wo)/Wo

where Ds is the degree of swelling and Wo and Wt are the weights of the samples in the dry and swollen states, respectively [8].

### 4.9. Cell Culture and Live/Dead Assay

AC16 Human CM cell lines (Millipore Sigma, Burlington, MA, USA) were cultured in complete growth medium and passaged for in vitro stabilization prior to their use in experiments. The complete growth medium consisted of Dulbecco’s Modified Eagle’s Medium/Nutrient Mixture F-12 Ham with 15mM HEPES and sodium bicarbonate and is liquid, sterile-filtered, and suitable for cell culture. To this, we added 12.5% fetal bovine serum, 1% penicillin-streptomycin, and 200 mM *L*-Glutamine. The cells were cultured, passaged, and stabilized for at least six passages before use in experiments, with the medium being changed every 24 h. At the end of every passage, normal and healthy cell morphology was confirmed using phase-contrast images. These were then seeded atop each gel sample at a density of 2 × 10^5^ cells/mL within 48-well plates and were incubated for 72 h (37 °C, 5% CO_2_), after which they were treated using a live/dead assay (Thermo Fisher Scientific, Waltham, MA, USA) for 1 h (37 °C, 5% CO_2_). This assay consisted of Calcein AM (green) that stained the live cells and Ethidium homodimer (red) that stained the dead cells, respectively. All live/dead stained cells were then imaged using confocal fluorescence microscopy (Olympus IX81 inverted fluorescence motorized microscope, Japan) to confirm the retention of viable cells within the samples and to detect the presence of dead cells present.

### 4.10. Statistical Analysis

All experiments were performed with sample groups of at least three or even more in some cases. Data are represented as the mean ± standard deviation (SD). Two-way ANOVA, followed by Tukey post-test for multiple comparisons, was performed to determine the statistical significance between individual sample groups with significance set at *p* < 0.05. 

## 5. Conclusions

Alginate hydrogels in tissue engineering and regenerative medicine have repeatedly demonstrated their functionality and usefulness for culturing of cells, among other applications. The intrinsic properties of the Alginate gel are what makes this material a viable primary phase for biocomposites in which a secondary phase such as PF108-SWCNT can enhance its mechanical and electronic properties to mimic endogenous tissue by reducing the disparity to the native mechanical characteristics of physiological tissues [4]. Among the studied surfactant-SWCNT groups, PF108-SWCNT pointed to a more consistent and comprehensive enhancement in efficiency to evenly disperse the HiPCo SWCNT in comparison to the other groups, as well as an overall increased performance when tested in contrast to control and other SWCNT-surfactant groups. These encouraging outcomes will lead to further research that may be necessary to comprehend thoroughly the enhanced electronic properties of the PF108-SWCNT hydrogel composite, as well as its applicability and optimization for 3D bioprinting and other biomedical applications beyond the scope of tissue engineering [48].

## Figures and Tables

**Table 1:** Summary of results. *p*-values indicated were obtained from two-way ANOVA followed by Tukey post-test for multiple comparisons were performed to determine the statistical significance between individual sample groups, and comparison of all SWCNT-surfactant-alginate with alginate controls. Results from comparisons having statistical significance (*p* < 0.05) were included.

Sample Description	Raman Spectra for SWCNT	NIR Spectra for SWCNT	Elastic Moduli & Complex Viscosity	Electrical Conductivity	Pore Size	Swelling and Degradation	Biocompatibility
SWCNT-PF108-alginate	Highest Signal Intensity	Highest Signal Intensity	Not statistically different compared to baseline	A significant increase compared to baseline *p* = 0.02 (*1); *p* = 0.02(*2)	Not statistically different compared to baseline	A significant decrease compared to baseline *p* = 0.03	Similar to the baseline
SWCNT-CTAB-alginate	Less compared to PF108	Less compared to PF108	Significant increase; *p* = 0.02 and *p* = 0.03, respectively	Not statistically different compared to baseline	Not statistically different compared to baseline	Not statistically different compared to baseline	NA
SWCNT-SDS-alginate	Less compared to PF108	Less compared to PF108	Not statistically different compared to baseline	Not statistically different compared to baseline	Not statistically different compared to baseline	Not statistically different compared to baseline	NA
Alginate	NA	NA	Baseline	Baseline	Baseline	Baseline	Baseline

NA: Not applicable, * indicates statistically significant difference (*p* < 0.05) for non-cross-linked (*1) and cross-linked samples (*2) of PF108, in comparison with controls.

## Supplementary Materials

Supplementary materials can be found at https://www.mdpi.com/1422-0067/20/19/4802/s1.

## Abbreviations

CNT	Carbon Nanotubes
SWCNT	Single-Walled Carbon Nanotubes
MWCNT	Multiwall Carbon Nanotubes
PF108	Pluronic F108
SDS	Sodium Dodecyl Sulfate
CTAB	Cetyltrimethylammonium Bromide
