# Supplementary material for: Fabrication of Surfactant-Dispersed HiPco Single-Walled Carbon Nanotube-Based Alginate Hydrogel Composites as Cellular Products
Source: Int J Mol Sci. 2019 Sep 27;20(19):4802. doi: 10.3390/ijms20194802 (PMC6801781; doi:10.3390/ijms20194802)
Supplement: Supplementary file 1 [file ijms-20-04802-s001.pdf]

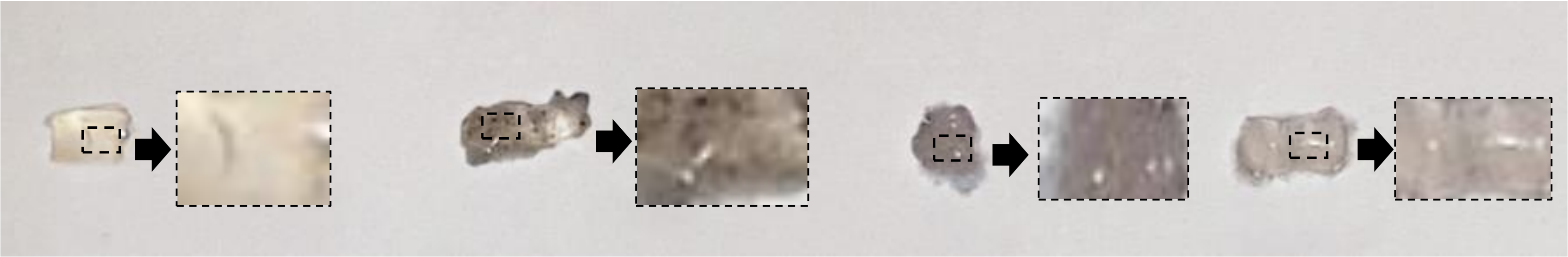

Control **CTAB** **SDS** **PF108**

Supplementary Figure 1. Representative images of control alginate gels (Control) and gels with SWCNT dispersed in CTAB, SDS and PF108 from left to right respectively.

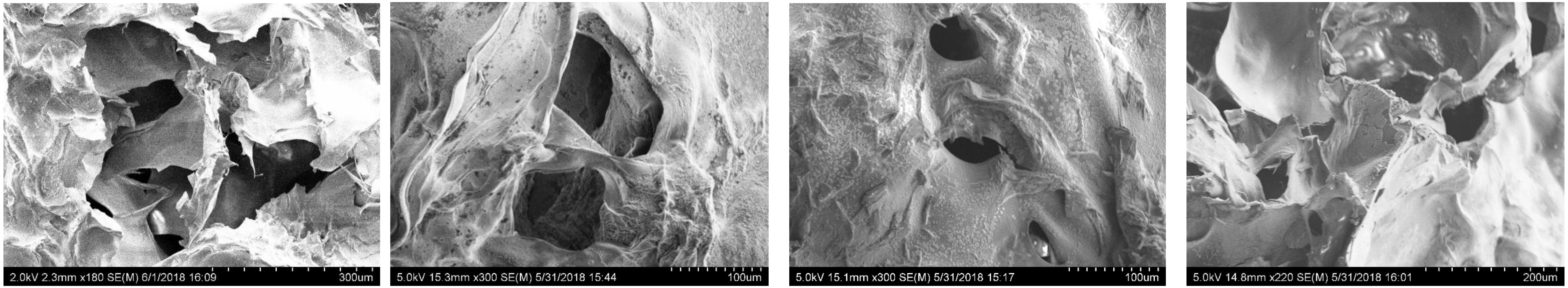

Control **CTAB** **SDS** **PF108**

Supplementary Figure 2. Characteristic SEM images
